# Supplementary material for: Predicting visual function by interpreting a neuronal wiring diagram
Source: Nature. 2024 Oct 2;634(8032):113–23. doi: 10.1038/s41586-024-07953-5 (PMC11446822; doi:10.1038/s41586-024-07953-5)
Supplement: Supplementary file 6 — CRF and ERF predictions for individual TmY4 and TmY9 cells. Analogous to Supplementary Data 3, but for TmY target types. Shown are the top four monosynaptic pathways, the strongest pathway passing through each of the top ten intermediary types (ranking from Extended Data Fig. 7), and the trisynaptic pathway Tm1–TmY–Dm3–TmY (see the section entitled Prediction of spatial normalization). [file 41586_2024_7953_MOESM6_ESM.zip › DataS4/TmY9q/720575940640886947.pdf]

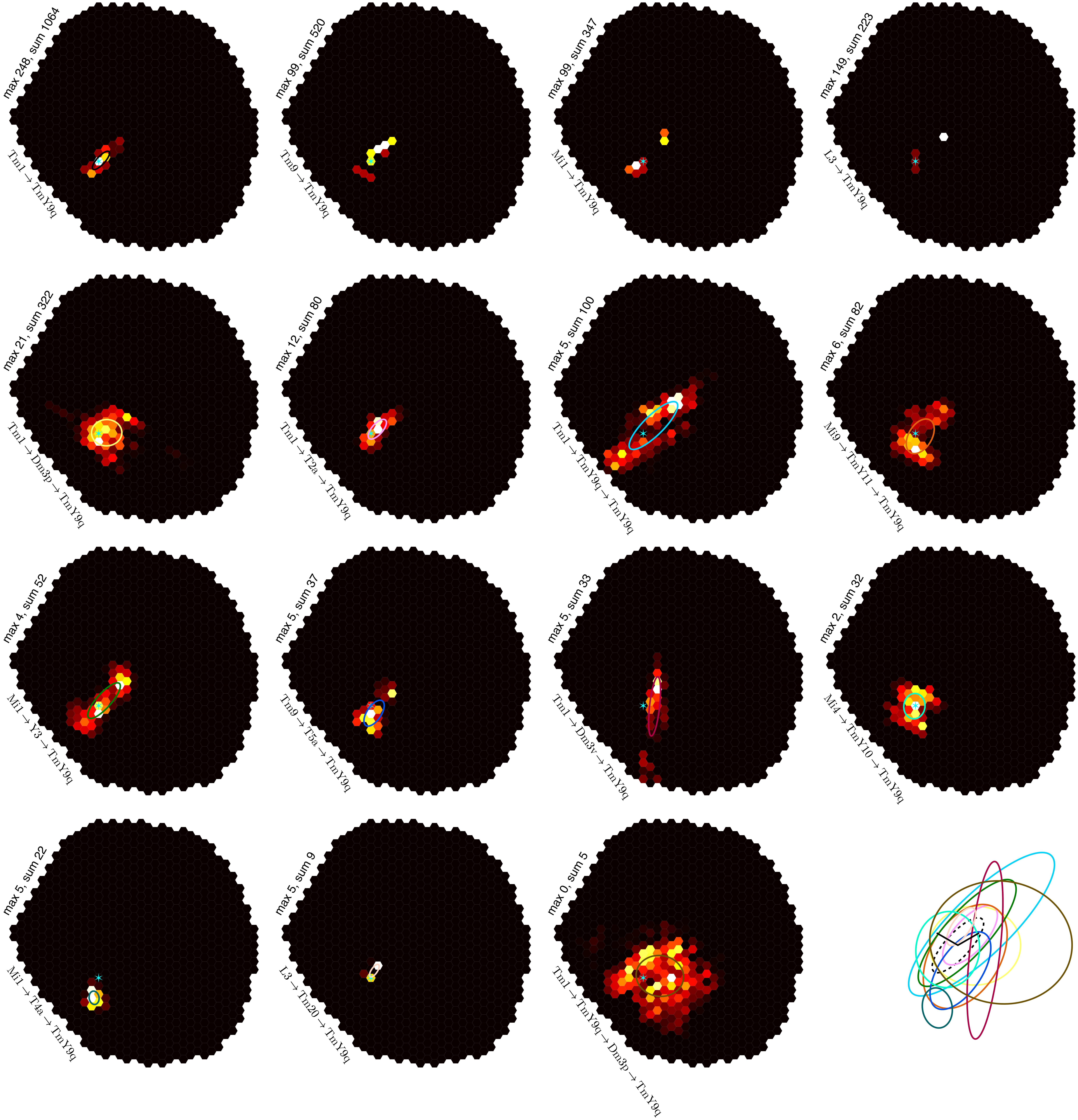
max 248, sum 1064
Tm1 → TmY9q
max 99, sum 520
Tm9 → TmY9q
max 99, sum 347
Mi1 → TmY9q
max 149, sum 223
L3 → TmY9q
max 21, sum 322
Tm1 → Dm3p → TmY9q
max 12, sum 80
Tm1 → T2a → TmY9q
max 5, sum 100
Tm1 → TmY9q → TmY9q
max 6, sum 82
Mi9 → TmY11 → TmY9q
max 4, sum 52
Mi1 → Y3 → TmY9q
max 5, sum 37
Tm9 → T5a → TmY9q
max 5, sum 33
Tm1 → Dm3v → TmY9q
max 2, sum 32
Mi4 → TmY10 → TmY9q
max 5, sum 22
Mi1 → T4a → TmY9q
max 5, sum 9
L3 → Tm20 → TmY9q
max 0, sum 5
Tm1 → TmY9q → Dm3p → TmY9q
